# Detecting chromosomal rearrangements in boars using Hi‐C

**DOI:** 10.1111/age.70009

**Published:** 2025-04-04

**Authors:** Frances Burden, Claudia Rathje, Peter Ellis, Justin Holl, Craig R. G. Lewis, Marta Farré

**Affiliations:** ^1^ School of Natural Sciences University of Kent Canterbury UK; ^2^ The Pig Improvement Company, Genus Plc Hendersonville Tennessee USA; ^3^ PIC Europe Sant Cugat del Valles, Barcelona Spain

**Keywords:** boar, chromosomal rearrangement, FISH, Hi‐C, reciprocal translocation

## Abstract

A chromosomal rearrangement such as a reciprocal translocation (RT) in a breeding boar can produce unbalanced gametes during meiosis, leading to a decreased litter size with detrimental economic implications for breeders. FISH and standard karyotyping are currently used to detect RTs, but a fresh sample is required, limiting the shipping conditions. Here, we investigated Hi‐C as an alternative technique to diagnose chromosome rearrangements. We show that Hi‐C can be used to detect such RTs from either a fresh or a frozen blood sample and therefore this technique represents an alternative to FISH for RT detection.

Pigs (*Sus scrofa*) account for over 30% of the global meat consumption, being one of the leading sources of meat protein worldwide and with a projected increase in demand (Food and Agriculture Organization report). Therefore, understanding and detecting the factors that lead to a decreased litter size (hypoprolificacy) is important. Among these factors are chromosomal abnormalities, particularly balanced reciprocal translocations (RTs). A reduction in litter size has been observed in boars carrying chromosomal abnormalities, particularly balanced RTs (Ducos et al., 2007; O’Connor et al., 2021; Sánchez‐Sánchez et al., 2019; Shams et al., 2021). Around 50% of hypoprolific boars are carriers of an RT, despite displaying a normal phenotype and good semen parameters (Quach et al., 2016). This occurs because RT carriers generate a high proportion of unbalanced gametes during meiosis. Moreover, 50% of the remaining gametes will be balanced RT carrier gametes, which produce surviving offspring that perpetuate the problem (Scriven et al., 1998). This results in the propagation of the RT across generations, with severe economic costs for pork production industries, ranging from £69 802 to £51 215 378 per undiagnosed RT carrier boar (Lewis et al., 2021).

Currently, inter‐chromosomal rearrangements can be detected by a number of different techniques such as karyotyping with G‐banding and FISH (O’Connor et al., 2017). Both karyotyping and FISH require fresh samples with cells actively dividing to obtain metaphase spreads. Moreover, karyotyping requires an experienced operator to identify small rearrangements, while FISH is less operator dependent, but its resolution is limited to approximately 5 Mb (Speicher & Carter, 2005) and a set of predetermined hybridization probes. Here, we studied whether Hi‐C, a new sequencing technique designed to detect the 3D structure of the nucleus, can be used to detect inter‐chromosomal rearrangements in boars, specifically RTs. Hi‐C was originally developed to reconstruct the 3D architecture of the genome (Lieberman‐Aiden et al., 2009) but it has been recently used in cancer research to identify small structural variants of around 1 Mbp (Harewood et al., 2017). Using animals previously diagnosed either as normal or as RT carriers by FISH, we assessed whether Hi‐C could be applied to screen for RTs in livestock species.

To do this, two animals from the same breed and breeding program were analysed using FISH and Hi‐C. FISH was performed as previously described (O’Connor et al., 2017), while the Arima Hi‐C+ kit (Arima Genomics) was used for Hi‐C (File S1).

First, using fresh blood samples, we established metaphase spreads and assessed whether animals were carriers of RTs using FISH (Figure 1a,b). One individual was diagnosed as normal (Figure 1a) and one a carrier of SSC1;2 RT (Figure 1a). Then, we investigated the effect of freezing samples on Hi‐C. To do so, we used the control sample and performed Hi‐C sequencing either fresh or after 1 week frozen at −20°C (Figure 1c,d). Using a HiCExplorer pipeline, we obtained 67 201 123 contacts for the fresh sample and 65 519 809 for the frozen sample. A total of 82 526 776 and 80 258 400 mappable read pairs were present for the fresh and frozen samples, respectively (Figure S1), with 26 785 085 and 30 971 242 inter‐chromosomal contacts detected in the fresh and frozen samples, respectively. After generating the contact matrices at 500 kbp resolution (File S1), no differences are observable between fresh and frozen samples (Figures 1c,d and 2a), indicating that freezing and thawing blood samples does not affect the broad 3D structure.

**FIGURE 1:**
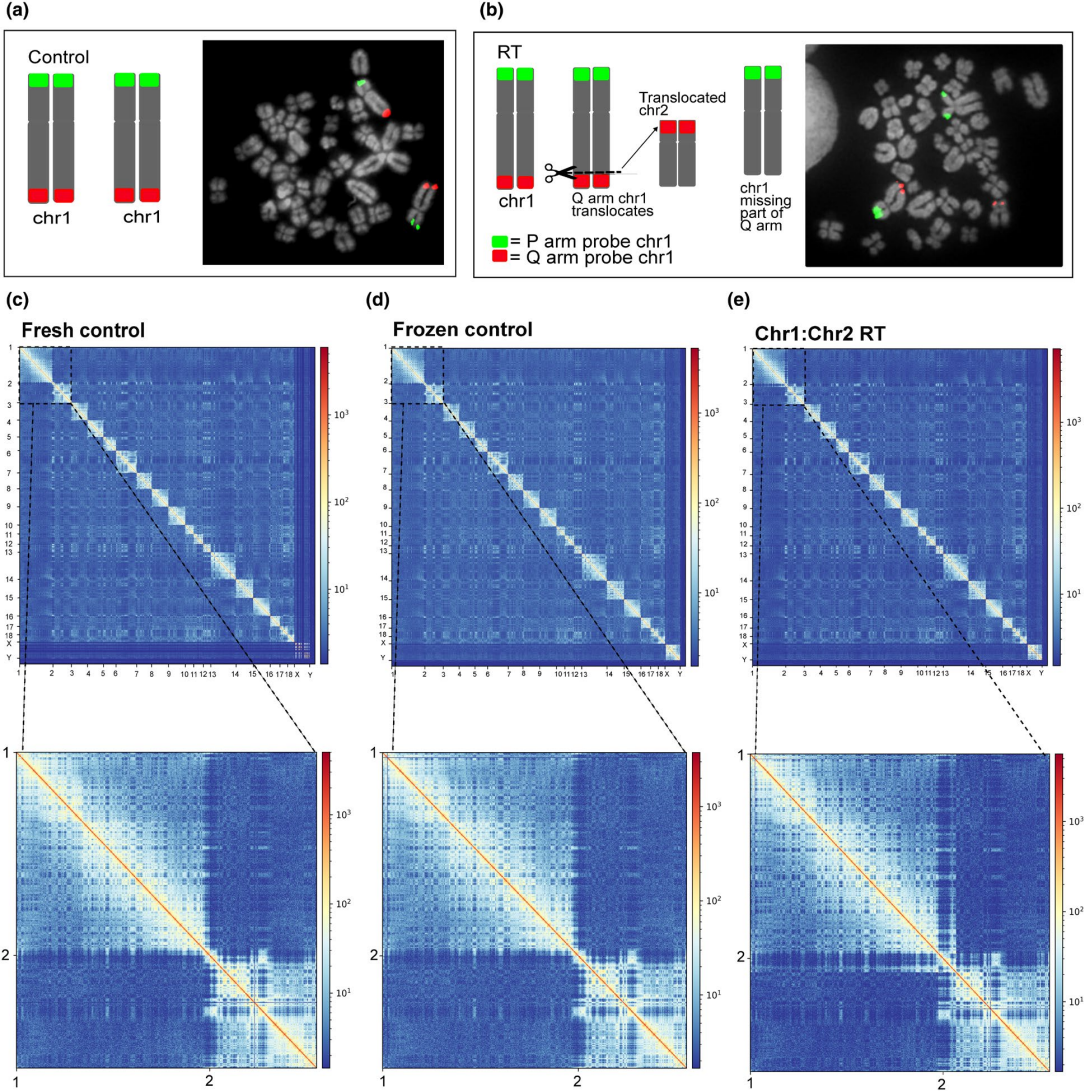
Diagnosis of control and reciprocal translocation (RT) samples using FISH (a, b). (c–e) Hi‐C contact matrixes at 500 kb resolution for a fresh control blood sample (c), a frozen control sample (d) and a chr1:Chr2 translocated sample (e), with zoom‐in panels for SSC1 and SSC2.

**FIGURE 2:**
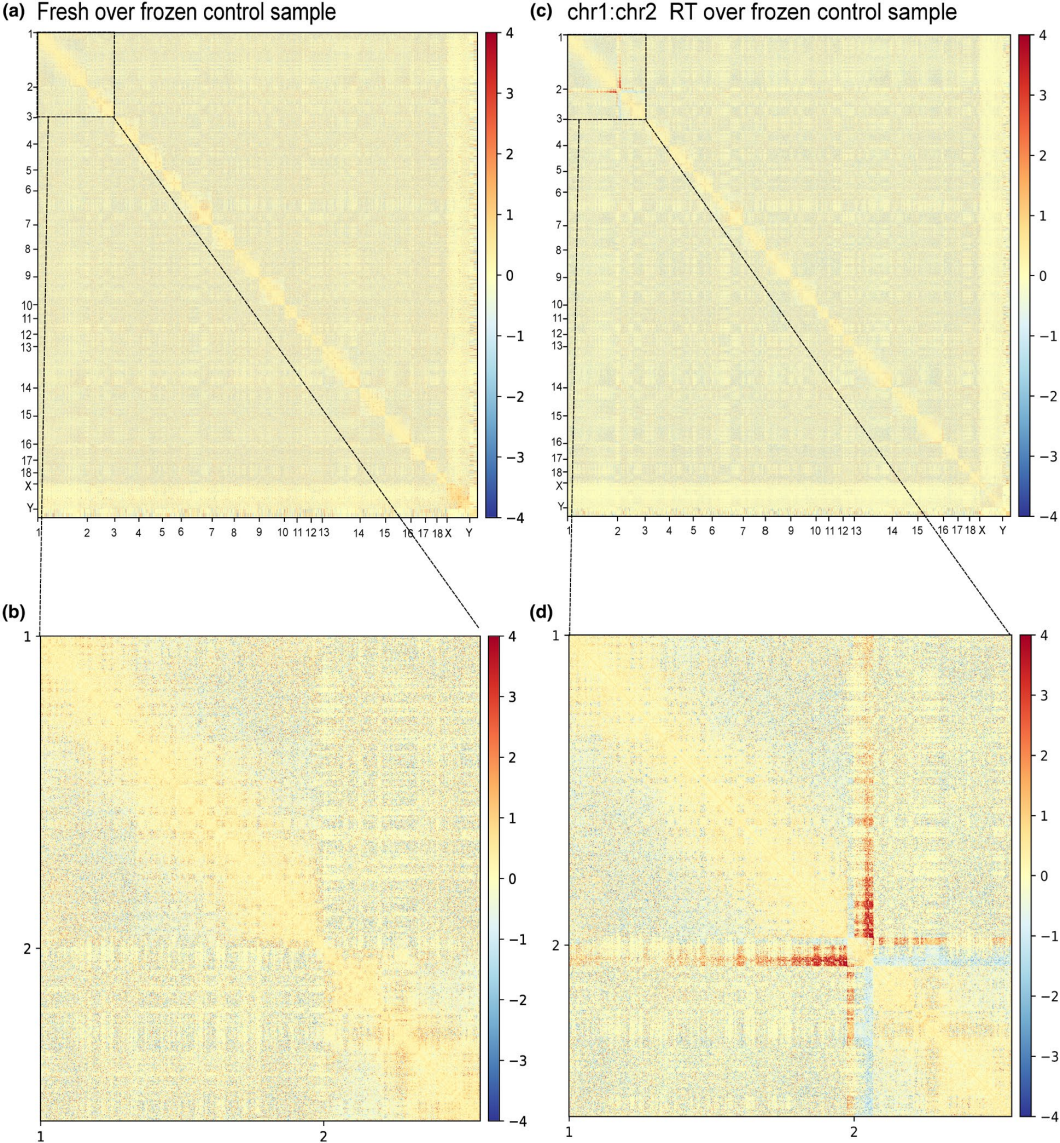
Comparison of Hi‐C matrices. (a) Comparison of the fresh and frozen samples and (b) zoom‐in for SSC1 and SSC2. (c) Comparison of reciprocal translocation (RT) and frozen samples, with zoom‐in for SSC1 and SSC2 (d). Log2ratio values are plotted.

We then performed Hi‐C sequencing on a frozen blood sample from the animal with a diagnosed RT (Figure 1b,e). We obtained 64 278 879 contacts and 84 990 099 mappable read pairs, similar to the control sample. At 500 kbp resolution, we found that when compared with the normal sample, a dramatic effect on the distribution of Hi‐C contacts in SSC1 and SSC2 chromosomes is seen (Figure 2b,d). An increase in contacts between SSC1qter and SSC2pter pinpoints the genomic region involved in the RT, in agreement with the FISH diagnosis.

Our results show that Hi‐C can be used to detect RTs in pigs, thus it could be used as an alternative to FISH to screen breeding boars for chromosomal abnormalities. Previous publications have shown that Hi‐C can detect unknown rearrangements (Harewood et al., 2017). If this RT was not previously known, at the current sequencing depth (of 100 million 150 bp reads) we would still reliably detect the translocation, as this sequencing depth should support a 40 kb resolution (Lajoie et al., 2014). Hi‐C does not rely on the presence of dividing cells; as such it can be used on all nucleated cell samples with minimal adjustments to the protocol. Since the cells do not need to be alive and culturable on receipt, they can be shipped frozen and fixed immediately on thawing. This is advantageous both from a logistical standpoint (multiple frozen samples can be batched together and processed simultaneously) and for biosafefy (freezing may inactivate pathogens). We have used blood for this study, but other tissue types would be suitable, provided 2.5–5 μg of DNA could be obtained. Although ~4× times more expensive than FISH, Hi‐C sequencing can be used to genotype and call all types of structural variants, including CNVs (Harewood et al., 2017), therefore making it a single solution for the breeding industry. Moreover, the ability to use frozen samples opens up new research opportunities utilising archived material from biobanks.

## AUTHOR CONTRIBUTIONS

**Frances Burden:** Investigation; writing – original draft; writing – review and editing; formal analysis; methodology; validation. **Claudia Rathje:** Investigation; validation. **Peter Ellis:** Writing – review and editing. **Justin Holl:** Resources. **Craig R. G. Lewis:** Resources. **Marta Farré:** Funding acquisition; writing – review and editing; conceptualization; supervision; project administration.

## FUNDING INFORMATION

MFB acknowledges support from the University of Kent's Biotechnology and Biological Sciences Research Council‐funded Impact Acceleration Account (BB/X511158/1).

## CONFLICT OF INTEREST STATEMENT

PE and CR run a service (CytoScreen Solutions) at the University of Kent, providing cytogenetic screening services to livestock breeding companies on a commercial basis.

## ETHICS STATEMENT

Blood samples were collected from boars undergoing routine genetic screening for chromosomal rearrangements. The samples were collected by in‐house trained veterinarians via standard phlebotomy. Following screening, remaining blood from animals found to have translocations (*n* = 1) and control animals (*n* = 1) was used for the current study.

## Supporting information


Data S1.

## Data Availability

Hi‐C contact matrices and ginteraction files are available at Zenodo https://doi.org/10.5281/zenodo.14849645.
