# Supplementary material for: Detecting chromosomal rearrangements in boars using Hi‐C
Source: Anim Genet. 2025 Apr 4;56(2):e70009. doi: 10.1111/age.70009 (PMC11969421; doi:10.1111/age.70009)
Supplement: Supplementary file 1 — Data S1. [file AGE-56-0-s001.docx]

**File. S1**

**Extended methods**

For Hi-C, 2 ml of fresh blood was mixed with 12 ml of ice-cold lysis buffer (155mM ammonium chloride, 10mM sodium bicarbonate, 127µM EDTA in deionized water) and incubated on ice and gently shaken every minute until the lysis was completed. The sample was spun at 1500 rpm for 5 min and the supernatant was discarded. This process was repeated once more. For frozen blood, the samples were thawed at 37 °C and then fixed without any red blood cell lysis.

A total of 0.4-2mls of fresh (red cell depleted) or frozen blood was fixed following the Arima Hi-C+ kit protocol (Arima Genomics). Aliquots of 0.5^e6^ fixed cells were pelleted and stored at –80 °C. One pellet of fixed cells was reverse cross-linked to check the yield of DNA. Cell pellets corresponding to 2-5µg of DNA were pooled and used as input for the Hi-C reaction, not exceeding a volume of 20µl. We followed the Arima Hi-C+ protocol except in increasing the conditioning step to 20 min and the enzyme digestion step to overnight. Proximity ligated DNA was sheared using a Diagenode Pico sonicator in 100µl of Arima elution buffer in 1.5ml LoBind tubes for 30 cycles of 30 seconds on, 30 seconds off, to achieve fragments of 200-800bp. Fragments <200bp were removed through size selection with AMPure XP Beads (Beckman Coulter) and biotin enrichment was performed. Library preparation and indexing followed Arima protocol. Libraries were quantified using a Qubit dsDNA broad range kit, diluted to 20ng/µl in nuclease free water and a 50µl aliquot was paired end sequenced at 150bp to obtain 30Gb of data (~100million reads).

Sequencing data quality was checked using FastQC (v0.11.9) (Andrews, 2020), and Trimmomatic (version 0.39) (Bolger, Lohse and Usadel, 2014) was used to remove reads with an average quality below 20. BWA-mem (0.7.17-r1198-dirty) with the options (-A1 -B4 -E50 –L0) was used to align read pairs independently to the pig reference genome (Sscrofa11.1) and *samtools view* (Li *et al.*, 2009) was used to convert the alignment to bam format. Alignment files for each individual pair were then merged using *samtools merge* and sorted (*samtools sort -n*).

HiCExplorer (3.7.4) (Wolff *et al.*, 2018, 2020) was used to generate Hi-C contact matrixes. First *hicFindRestSite* was used to find restriction enzyme sites within the pig genome with two *searchPattern GATC* and *GA.TC*. We generated h5 matrices at 500kb resolution using HicBuildMatrix, with *--restrictionSequence* GATC GA.TC *--danglingSequence* GATC A.TC. Quality metrics were computed with HicBuildMatrix and hicQC and plotted using ggplot2. We then normalised all libraries using hicNormalise with –normalize smallest across all samples. Normalised data was then used for all subsequent plotting. hicCorrectMatrix *diagnostic_plot* was used to determine the thresholds for matrix correction and hicCorrectMatrix was then run with upper threshold of 5 and the lower threshold between -2.2 to -7.

Finally, to compare and plot matrices, we used hicCompareMatrices and hicPlotMatrix with default parameters.

Fig.S1: Mapping statistics for the fresh (shown in blue) and the frozen (shown in orange) Hi-C sample.


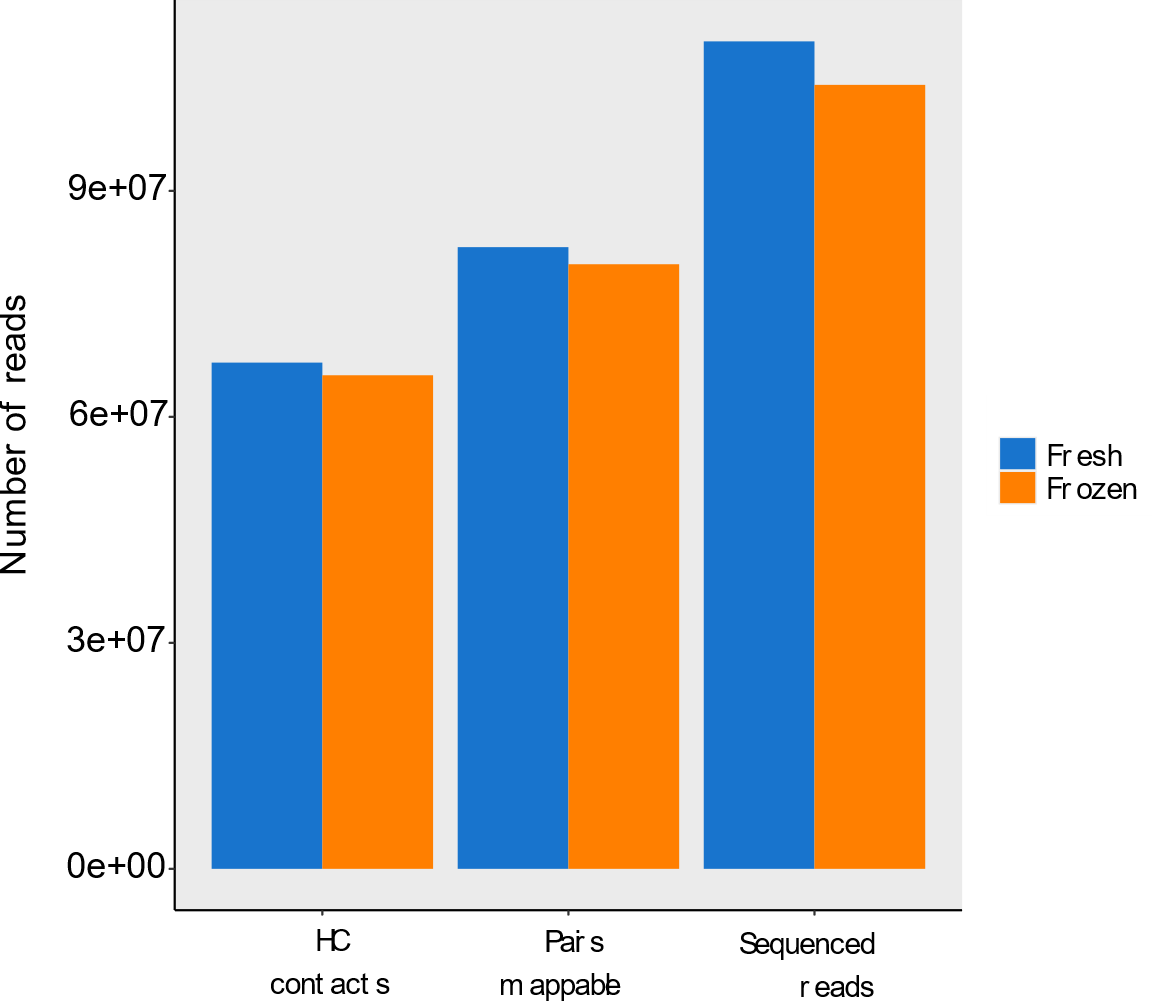

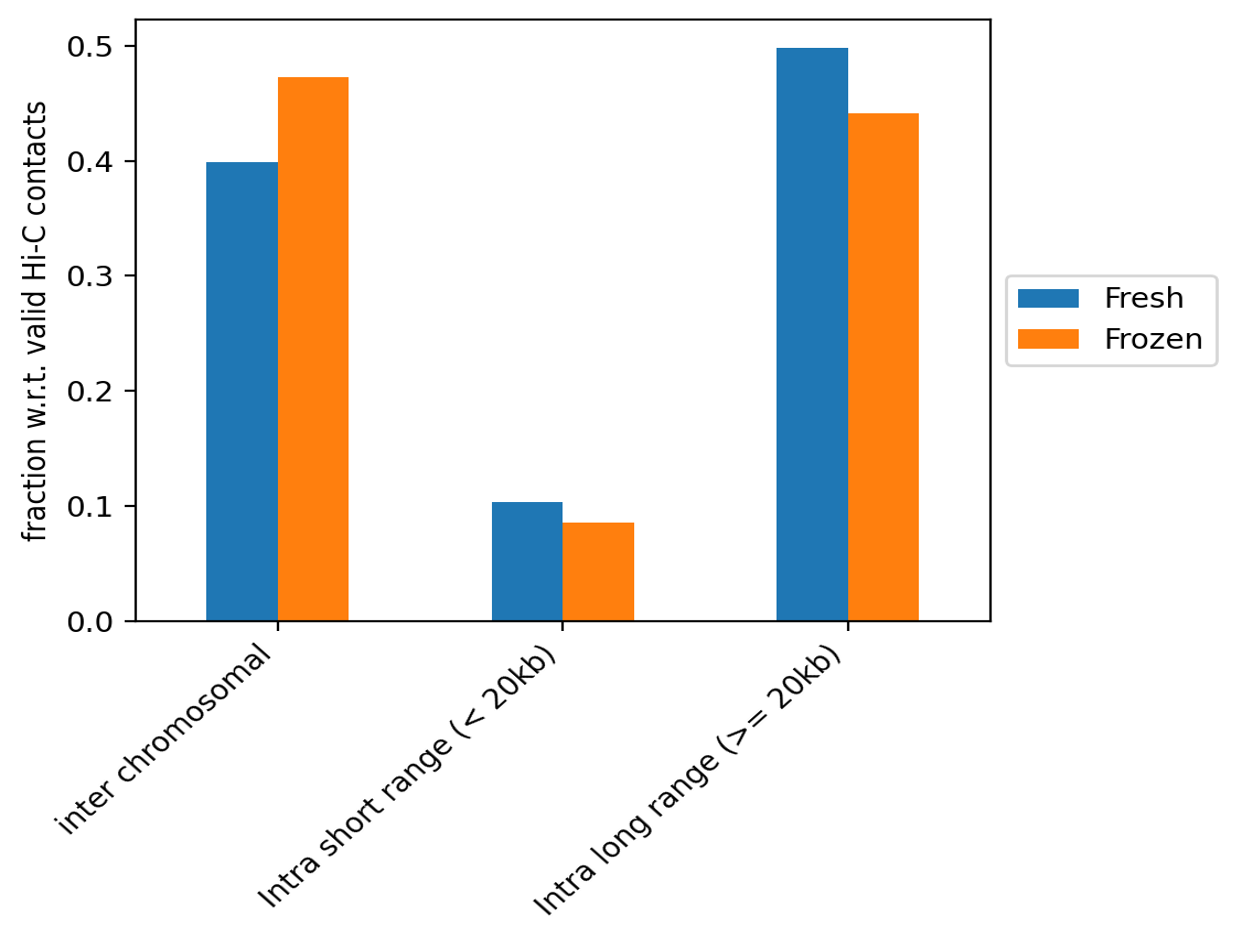


**References**

Andrews, S. (2020) ‘ FastQC: a quality control tool for high throughput sequence data’.

Bolger, A.M., Lohse, M. and Usadel, B. (2014) ‘Trimmomatic: a flexible trimmer for Illumina sequence data’, *Bioinformatics (Oxford, England)*, 30(15), pp. 2114–2120. Available at: https://doi.org/10.1093/BIOINFORMATICS/BTU170.

Li, H. *et al.* (2009) ‘The Sequence Alignment/Map format and SAMtools’, *Bioinformatics (Oxford, England)*, 25(16), pp. 2078–2079. Available at: <https://doi.org/10.1093/BIOINFORMATICS/BTP352>.

Wolff, J. *et al.* (2018) ‘Galaxy HiCExplorer: a web server for reproducible Hi-C data analysis, quality control and visualization’, *Nucleic Acids Research*, 46(Web Server issue), p. W11. Available at: <https://doi.org/10.1093/NAR/GKY504>.

Wolff, J. *et al.* (2020) ‘Galaxy HiCExplorer 3: a web server for reproducible Hi-C, capture Hi-C and single-cell Hi-C data analysis, quality control and visualization’, *Nucleic acids research*, 48(W1), pp. W177–W184. Available at: <https://doi.org/10.1093/NAR/GKAA220>.
